# Smallpox Zero: An Illustrated History of Smallpox and Its Eradication

**DOI:** 10.3201/eid1611.101145

**Published:** 2010-11

**Authors:** P. Lynne Stockton

**Affiliations:** Author affiliation: Centers for Disease Control and Prevention, Atlanta, Georgia, USA

**Keywords:** Smallpox, eradication, viruses, history, book review

Smallpox Zero is an excellent commemoration of the 30th anniversary of smallpox eradication. This picture book tells the story of the disease from its origin through eradication and beyond (proposed fate of virus stock). The story is presented in short bubbles of text on a background of colorful, hand-drawn pictures. The children’s book packaging (8½″ × 11″ × ¼″ with glossy, illustrated cover) belies the large amount of well-organized information inside. The 10 chapters draw you in with enticing titles such as No One Escaped. Once you’re inside, the artwork captivates you. Practically every drawing would be suitable for framing and display. The maps, landscapes, and faces bring humanity’s greatest scourge back to life for readers.

But who are the intended readers? The design and layout seem aimed at youth. A busy background with multiple drawings is right on target for teens, and the drawings are in the style of youth-oriented action comics. Even the need to reach youth is explained in the Note to Readers, “Since the battle against it [smallpox] was waged in remote areas far from the gaze of television cameras, the younger generations may have difficulty in appreciating the nature and magnitude of the task of global eradication.”

The youth-attracting design, however, is mismatched with complex content. Readability scales rank average reading level at grade 12; several segments rank higher than grade 14. (For comparison, the reading level of this review is grade 11.) Multisyllabic words such as disease “transmission” rather than “spread” are the culprits. Adding to the complexity is mention of places and populations that young readers probably never heard of, like Sarawak and Hittites.

Readers can at least find many terms defined in a glossary in the back of the book. However, use of boldface or color to indicate which terms are defined would have been helpful. Today’s impatient readers, accustomed to the instant gratification of online pop-up bubbles, may be unwilling to spend time flipping pages to check. Also helpful would have been a table of contents and index.

Audience and navigation tools aside, this is still a powerful book that should preserve the memory of smallpox and its heroes for future generations. Jonathan Roy, illustrator and narrator, effectively offers something for everyone, regardless of their location, education level, and native language. The global, rather than country-centric, perspective should appeal to readers worldwide; the small bites of information make each message palatable for even low-literacy readers; and the drawings eliminate language barriers. All these factors work together to increase access to the message that we should not take our freedom from smallpox for granted.

Although I cannot see Smallpox Zero being studied in a classroom, I can easily see it being browsed in a reception room, a reading room, a waiting room, or even a living room. A quick glance is enough to create an overall appreciation for the millions worldwide who suffered from smallpox and for the dedicated and selfless men and women we have to thank for its eradication.

